# Non‐genetic heterogeneity, altered cell fate and differentiation therapy

**DOI:** 10.15252/emmm.202012670

**Published:** 2021-02-08

**Authors:** Alexander C Lewis, Lev M Kats

**Affiliations:** ^1^ The Peter MacCallum Cancer Centre Melbourne VIC Australia; ^2^ The Sir Peter MacCallum Department of Oncology University of Melbourne Parkville VIC Australia

**Keywords:** cancer, cell fate, differentiation, differentiation therapy, self‐renewal, Cancer, Haematology, Regenerative Medicine

## Abstract

Altered capacity for self‐renewal and differentiation is a hallmark of cancer, and many tumors are composed of cells with a developmentally immature phenotype. Among the malignancies where processes that govern cell fate decisions have been studied most extensively is acute myeloid leukemia (AML), a disease characterized by the presence of large numbers of “blasts” that resemble myeloid progenitors. Classically, the defining properties of AML cells were said to be aberrant self‐renewal and a block of differentiation, and the term “differentiation therapy” was coined to describe drugs that promote the maturation of leukemic blasts. Notionally however, the simplistic view that such agents “unblock” differentiation is at odds with the cancer stem cell (CSC) hypothesis that posits that tumors are hierarchically organized and that CSCs, which underpin cancer growth, retain the capacity to progress to a developmentally more mature state. Herein, we will review recent developments that are providing unprecedented insights into non‐genetic heterogeneity both at steady state and in response to treatment, and propose a new conceptual framework for therapies that aim to alter cell fate decisions in cancer.

## Graphical abstract

This review by A. Lewis and L. Kats summarizes the recent advances in our understanding of "differentiation therapy". The authors share new developments into non‐genetic heterogeneity, and propose a conceptual framework for therapies that aim to alter cell fate decisions in cancer.

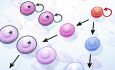

**Glossary**
Cellular plasticity: The capacity of a cell to reprogram its transcriptional and epigenetic state and adopt a new phenotype.
Cellular hierarchy: In some tissues, cells are hierarchically organized. Cells at the top levels (e.g., stem or multi‐potent progenitor cells) can give rise to cells at lower levels (e.g., restricted progenitors or effector cells), but not vice versa.
Cancer stem cell (CSC): A type of malignant cell is capable of limitless self‐renewal and is thought to be responsible for tumor maintenance and propagation. CSCs share many features with normal stem cells. In the context of leukemia, CSCs are referred to as leukemic stem cells (LSCs).
De‐differentiation: Conversion of a mature or terminally differentiated cell to a stem‐like cell.
Differentiation: The process by which the features, function, or potential of a cell is altered, e.g., conversion of a multi‐potent stem cell to a lineage‐committed progenitor. Differentiation is underpinned by alterations in the chromatin and transcriptional states of the cell.
Differentiation arrest: A phenomenon whereby cells are not able to follow their normal maturation to become a terminally differentiated and fully functional effector cell.
Differentiation therapy: A therapeutic strategy that induces the conversion of cellular states. In the context of cancer, differentiation therapy is intended to convert cells from a self‐renewing to a non‐self‐renewing phenotype.
Differentiation trajectory: The development and maturation of a cell over time.
Epigenetic memory: A phenomenon where daughter cells inherit information from the mother cell following cell division. Epigenetic memory is controlled by chromatin regulators that enable and enforce selective gene expression.
Exhaustion: Loss of self‐renewal capacity due to constant proliferation and/or differentiation.
Hematopoiesis: The process of normal blood cell development.
Lineage bias: A phenomenon where a cell with multi‐potent capabilities preferences a particular fate over others, e.g., in the hematopoietic system, some hematopoietic stem cell subsets generate reduced numbers of lymphoid cells (myeloid biased) whereas others generate fewer myeloid cells (lymphoid biased).
Non‐genetic heterogeneity: Also known as epigenetic heterogeneity. Genetically clonal cells can exhibit variable phenotypes, functions, and fates. The variation is controlled by both cell intrinsic (e.g., chromatin state) and cell extrinsic (e.g., position within the tumor) factors.
Self‐renewal: A type of mitotic event where the two daughter cells have the same features, function, and potential as the mother cell.
Stem cell: A cell that is essential for the maintenance of a particular tissue. In some tissues, such as the hematopoietic system, stem cells are rare specialized cells capable of self‐renewal and multi‐lineage differentiation (i.e., the development of many different cell types). In other contexts, many cells can behave as a stem cell and can contribute to tissue regeneration. Stem cell activity is assessed *in vivo*, typically using lineage tracing of transplantation. In the hematopoietic system, stem cells are referred to as hematopoietic stem cells (HSCs).

## Introduction

During normal development and under homeostatic conditions, mammalian tissues are hierarchically organized with long‐lived stem cells maintaining a repertoire of effector cells through a carefully calibrated balance between self‐renewal and differentiation. Cells follow prescribed trajectories involving proliferation, specification, and death, ensuring that appropriate numbers and ratios of the various cell types are preserved. At any given juncture, the phenotype and fate of an individual cell is dictated by its epigenetic state, enabling the generation of the myriad specialized cells required for the existence of a multi‐cellular organism from a single genome.

In cancer, well‐established lineage paths are perturbed resulting in over‐production of morphologically abnormal dysfunctional cells. As early as the 1970s, scientists noticed that certain cytokines and other signaling molecules could be deployed *in vitro* not only to arrest the growth of cancer cells, but indeed to convert them to what appeared to be “normal” non‐malignant cells (Strickland & Mahdavi, 1978; Breitman *et al*, 1981; Sidell, 1982). These initial pioneering observations led to the idea for a fundamentally different approach to cancer treatment—differentiation therapy. Rather than seeking to kill cancer cells, differentiation therapies aim to reprogram corrupted cells to resume their ordinary developmental trajectory, leading to maturation and elimination by natural clearance mechanisms. Differentiation therapies offer an attractive paradigm in the context of cancer, promising lower side effects as well as extermination of rare cancer stem cells (CSCs) that underpin therapy resistance and disease relapse following cytotoxic drugs (de Thé, 2018; Lin, Srikanth *et al*, 2018). Conversely, the ability of cells to adopt new cellular states in response to environmental challenge, referred to as cell fate plasticity, is emerging as an important drug resistance mechanism (Bell & Gilan, 2020).

With the advent of single‐cell technologies, we now have the opportunity to define transcriptional, epigenetic, and signaling features that regulate developmental decision‐making at unprecedented resolution. In this review, we highlight recent advances in our understanding of normal and malignant differentiation. We focus initially on hematopoiesis and acute myeloid leukemia (AML) as model systems where knowledge of molecular programs that dictate cell phenotype and fate is most advanced. We summarize recent data that demonstrate that altered cell fate and epigenetic reprogramming underpin response and resistance to a growing number of therapies. We discuss the interplay between epigenetic heterogeneity, genetic heterogeneity, and the immune system. Finally, we speculate how increased knowledge of the mechanistic basis of existing drugs can be leveraged to develop next‐generation anti‐cancer modalities.

## Normal blood development—the challenge of maintaining the masses

Before embarking on a discussion of malignant development, it is worth considering normal developmental trajectories. One of the best studied developmental systems is hematopoiesis, which has long served as a model for hierarchically organized tissues (Laurenti & Göttgens, 2018). Situated at the apex of the hematopoietic hierarchy are hematopoietic stem cells (HSCs), uniquely endowed with the capability to maintain blood cell production from development to death. Classically, HSCs are defined as possessing two essential characteristics: (i) long‐term self‐renewal—the capacity to generate more HSCs over the life span of the animal and (ii) multi‐lineage differentiation—producing cells of the myeloid, lymphoid, erythroid, and megakaryocytic lineages. The HSC reservoir must remain finely poised, providing sufficient differentiated progeny to meet changing requirements on the one hand, while maintaining longevity and avoiding exhaustion on the other. To balance these competing tasks, HSCs must be capable of generating progeny of unequal potential, although the process by which this occurs remains contentious (Loeffler and Schroeder (2019). Elegant studies from multiple groups have observed individual mitotic events *in vitro* and *in vivo*, and identified various factors that are linked with either self‐renewal or differentiation of the daughter cells (Ito *et al*, 2012; Ting *et al*, 2012; Ito *et al*, 2016; Loeffler *et al*, 2019). However, whether distinct patterns of inheritance in newly formed cells represent *bona fide* asymmetric cell division that prospectively regulates cell fate (e.g., via unequal distribution of transcription factors), or is acquired shortly after but independent of mitosis (e.g., via stochastic transcriptional events or unequal exposure to extrinsic signals), has not been fully resolved.

Downstream of HSCs, differentiation through the hematopoietic hierarchy requires cells to transit through multiple states, gradually shutting down self‐renewal and multi‐lineage capabilities while upregulating specific effector functions. Cells at the intermediate levels of the hierarchy act as an amplifier, allowing vast numbers of mature cells to be created from a minute pool of HSCs that rarely divide. It has been estimated that humans possess approximately 10^3^–10^4^ HSCs that are capable of generating in the order of 10^14^ mature blood cells per year (Catlin *et al*, 2011). As cells progress along their developmental trajectory, lineage‐defining transcription factors (TFs) recruit epigenetic regulators to progressively remodel the chromatin state, driving gene expression programs specific to particular cell types (Bock *et al*, 2012; Lara‐Astiaso *et al*, 2014; Buenrostro *et al*, 2018). Complex autoregulatory and cross‐antagonistic TF circuits enable cells to “choose” between alternative paths, simultaneously stabilizing a particular fate while suppressing others. Thus, the myeloid TF PU.1 positively regulates its own expression while suppressing the erythroid TF GATA1 and vice versa (Nerlov *et al*, 2000; Zhang *et al*, 2000; Hoppe *et al*, 2016); the monocyte TF IRF8 competes with the neutrophil TF GFI1 (Olsson *et al*, 2016); and RUNX1 and RUNX3 co‐operate to ensure commitment to CD8^+^ over CD4^+^ T‐cell identity (Hsu *et al*, 2016). The interplay between TFs and chromatin‐regulating factors enables “epigenetic memory” ensuring that cell fate information is transmitted during mitosis and maintained in subsequent cell generations.

Over the past decade, technological advances in omics, lineage tracing, and single‐cell methodologies have led to an evolving model of hematopoiesis as a continuum of hierarchically organized populations rather than a rigid “branching tree” structure. Early studies used flow cytometry to isolate cell “types”, the potential of which was then functionally analyzed by transplantation. While this approach revolutionized our understanding of blood development, it was limited by its dependence on a small number of cell surface markers, as well as the necessity to classify any given cell as belonging to a single sub‐population. However, as more and more features of individual cells could be simultaneously measured, it became apparent that classically defined sub‐populations previously thought to be uniform and distinct were actually heterogeneous and partially overlapping (Velten *et al*, 2017; Giladi *et al*, 2018). Moreover, we now appreciate that the output of stem and progenitor cells at steady state and under stress conditions (as is the case during transplantation) are vastly different (Pei *et al*, 2017). Put another way, in most cases cell fate is a subset of cell potential. Collectively, these studies point to a system where gradual progression between states enables plasticity at multiple levels of the hierarchy and thereby the capacity to rapidly adapt to changing requirements of the organism.

## AML—a disease of altered cell fate

**Figure 1:**
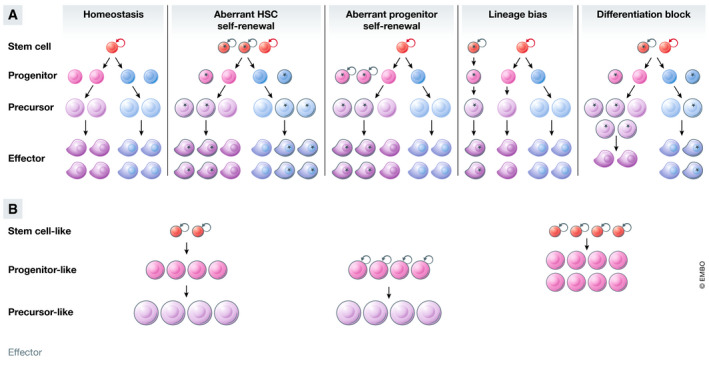
Genetic mutations disrupt cell fate and alter cellular hierarchy (A) During normal development, hematopoiesis is hierarchically organized. Stem cells at the apex of the hierarchy self‐renew or differentiate into effector cells in a stepwise process in a unidirectional manner. Recurrent mutations in cancer can alter multiple facets of cell fate (mutant cells indicated with a black outline). Stem cells or lineage‐committed progenitors can acquire aberrant self‐renewal, resulting in increased frequency and contribution to more differentiated cell types. The output of multi‐potent stem or progenitor cells can be biased toward a particular lineage. Cells at various stages of the hierarchy can become blocked in differentiation, unable to mature to form fully functioning effector cells. (B) Acute myeloid leukemia (AML) cells are similarly organized in a hierarchy, albeit a truncated one. Gene expression in AML cells resembles expression in their normal counterparts to a large degree, so much so that the AML hierarchy can be “projected” onto the normal hematopoietic hierarchy. Hierarchies from different AML patients contain different numbers of the various cell types depending on the underlying driver mutations, and the cell type and sequence in which they occurred.

Acute myeloid leukemia is a devastating malady with a 5‐year overall survival rate that still languishes below 30%. In 2002, at the dawn of the genomics era Gilliland and Griffin proposed a two‐hit model of leukemogenesis (Gilliland & Griffin, 2002). In their model, two genetic lesions were required to initiate AML—a class I mutation that confers proliferation and aberrant self‐renewal; and a class II mutation that establishes the block of differentiation. While we now know that most AML patients carry more than two driver mutations and that individual mutations cannot be neatly characterized into class I or class II (The Cancer Genome Atlas, 2013; Papaemmanuil *et al*, 2016), the basic premise that AML results from dysregulated developmental processes holds true (Fig 1A).

As is the case with normal blood cells, genetically clonal AML cells are not homogenous but exist within a developmental hierarchy, albeit a truncated one (Fig 1B). Among the first to propose the existence of a hierarchy in cancer were Bonnet and Dick, who demonstrated that AML cells expressing HSC markers had increased capacity to initiate disease when transplanted into immune compromised mice (Bonnet & Dick, 1997). Many subsequent studies have reproduced these initial observations in various contexts and confirmed that genetically clonal AML cells are indeed phenotypically and functionally heterogeneous (Bonnet & Dick, 1997; Gilliland & Griffin, 2002; Krivtsov *et al*, 2006; Quek *et al*, 2018; van Galen *et al*, 2019). Only a fraction of leukemic cells, termed leukemia stem cells (LSCs, equivalent to CSCs in solid tumors), have the capability to sustain tumor growth long term. LSCs are typically rare, quiescent, and analogous to HSCs, able to both self‐renew and differentiate to generate “bulk” tumor cells with limited proliferative capacity (Bonnet & Dick, 1997; Boyd *et al*, 2018). LSC frequency has been linked with aggressive disease progression, drug resistance, and relapse (Ng *et al*, 2016), although recent studies have suggested that features other than quiescence may be important in the context of chemotherapy resistance (Farge *et al*, 2017; Boyd *et al*, 2018). In particular, the specific metabolic state of LSCs, namely high oxidative phosphorylation fueled by increased amino acid metabolism and fatty acid synthesis, appears to play a key role in the ability of LSCs to withstand chemotoxic stress (Lagadinou *et al*, 2013; Jones *et al*, 2018).

Although AML mimics the hierarchical structure of normal hematopoiesis, the developmental topologies are highly varied between different AML patients and model systems (Fig 1B). Functionally defined LSCs express different surface markers depending on factors such as the underlying genetic lesion (Zhou & Chng, 2014; Pabst *et al*, 2016; Ketkar *et al*, 2020). The situation is further complicated when defining human LSC activity due to the limitations of xenotransplantation (and the differential capacity of particular cell types to engraft in certain strains of immune compromised mice), with more recent studies highlighting the previous underestimation of LSC frequency derived from earlier models (Klco *et al*, 2014; Reinisch *et al*, 2016). Transcriptionally, cells at the apex of the malignant hierarchy can most closely resemble HSCs or committed myeloid progenitors; likewise, the developmental arrest can occur either early or late along the myeloid differentiation trajectory (Bonnet & Dick, 1997; Gilliland & Griffin, 2002; Quek *et al*, 2018; van Galen *et al*, 2019) (Fig 1B). Interestingly, individual leukemic cells can exist in “mixed” cellular states, simultaneously possessing features that normally occur in different cell types in non‐malignant cells (Corces *et al*, 2016).

Mouse genetic studies over the past two decades have been critical in delineating the contribution of recurrent driver mutations to altered differentiation during leukemia initiation and maintenance. The effects of individual lesions on cell fate decisions, and consequently the number and type of cells produced from the mutant clone, are complex and varied (Fig 1A). These have been reviewed comprehensively elsewhere (Kishtagari *et al*, 2020), but we provide some examples here to illustrate general principles. Mutations in *DNMT3A* and *TET2* increase HSC self‐renewal, providing mutant cells with a competitive advantage over their wild‐type counterparts (Moran‐Crusio *et al*, 2011; Challen *et al*, 2012). These mutations are highly enriched in a condition termed clonal hematopoiesis of indeterminate potential (CHIP), demonstrating that the phenotype is conserved between mice and humans (Jaiswal *et al*, 2014). Alternations in *KMT2A*, *NPM1,* and *BCOR* endow committed myeloid progenitors with self‐renewal capabilities, in part by re‐instating HSC‐associated transcriptional programs (Krivtsov *et al*, 2006; Kelly *et al*, 2019; Uckelmann *et al*, 2020). Mutations in *IDH1*, *IDH2*, *JAK2,* and *CEBPα* block differentiation and alter lineage bias of various progenitor populations (Zhang *et al*, 2004; Mullally *et al*, 2010; Sasaki *et al*, 2012; Kats *et al*, 2014).

**Figure 2:**
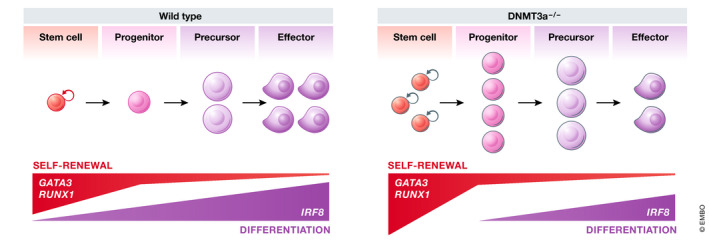
AML driver mutations have complex effects on gene expression and cell fate Individual mutations induce cell context‐dependent gene expression changes and alter cell fate at multiple points along the differentiation trajectory. As an example, loss of *DNMT3A* results in increased expression of self‐renewal‐associated genes *GATA3* and *RUNX1* in stem and progenitor cells, but not in more differentiated cells; conversely, the expression of the lineage‐associated transcription factor *IRF8* is upregulated specifically in more mature cells. As a consequence, maturation of *DNMT3A* knockout cells is altered, resulting in accumulation of some developmental cell types and depletion of others (Challen *et al*, 2012; Ketkar *et al*, 2020).

Individual lesions can perturb the hierarchy at multiple points, increasing the production or maintenance of some cell types, but decreasing others. As an example, *DNMT3A* plays distinct compartment‐specific roles in fate decisions, affecting the phenotypes of both HSCs and committed myeloid progenitors (Izzo *et al*, 2020; Ostrander *et al*, 2020) (Fig 2). Likewise, *TET2* knockout alters self‐renewal of HSCs, skews differentiation in favor of the myelomonocytic lineage over the erythroid, and alters B‐cell differentiation (Dominguez *et al*, 2018; Ito *et al*, 2019; Izzo *et al*, 2020). Interestingly, even mutations in the same gene can result in different outcomes. Loss of TET2 expression affects both myeloid and lymphoid differentiation, whereas mutations in the TET2 catalytic site affect the former but not the latter (Ito *et al*, 2019). Similarly, the phenotypes induced by *DNMT3A* nonsense and missense mutations are markedly different as evidenced from both mouse modeling studies and single‐cell DNA sequencing analyses of patient samples (Guryanova *et al*, 2016; Ketkar *et al*, 2020; Miles *et al*, 2020). Collectively, these observations explain how individual genes can simultaneously play oncogenic and tumor‐suppressive roles in different blood cancers, and why mutational patterns in genes such as *IDH1/2*, *JAK2, EZH2,* and *DNMT3A* vary across disease settings.

At the molecular level, the complex phenotypes that result from a single genetic event are explained by the context‐dependent activity of individual proteins (Fig 2). Proteins function in a complex cellular milieu that is different even between closely related cell types. For a given protein, the relative expression of its upstream regulators, co‐factors, and downstream targets vary at different developmental stages. As a result, epigenetic and transcriptional perturbations caused by genetic changes are highly cell type specific. The RUNX1‐ETO fusion protein that results from the t(8;21) chromosomal translocation found in 5–10% of AML patients antagonizes the activity of wild‐type RUNX1 encoded by the remaining unaffected allele. An elegant study from Constanze Bonifer and colleagues (Regha *et al*, 2015) compared the transcriptional response of hematopoietic cells to the induction of RUNX1‐ETO at early and late stages of maturation, and found that the induction and repression of target genes was different between the two developmental contexts. Similarly, the DNA regions that are differentially methylated following loss of *DNMT3A* or *TET2* are different between different cell types (Kaasinen *et al*, 2019; Ketkar *et al*, 2020). These studies have been reaffirmed more recently by the application of single‐cell RNA sequencing (scRNAseq) (Cheng *et al*, 2019; Izzo *et al*, 2020) and underscore the importance of considering context when seeking to identify regulators and mediators of oncogenes and tumor suppressors.

As a result of the dynamic interaction between genetic and epigenetic changes during leukemogenesis, the differentiation hierarchy of an AML clone and the behavior of individual leukemic cells are dictated not only by the nature of genetic events, but also the developmental stage in which they occur. Mutations arise in a so‐called “cell of origin”, and not surprisingly, the established chromatin state of that cell can impact on the ability of a particular driver mutation to reprogram the transcriptome. A case in point is *MLL*‐rearranged AML. In mice, MLL fusion proteins can induce aberrant self‐renewal when introduced into either HSCs or committed myeloid progenitors. While in both cases, cells transplanted *in vivo* can initiate leukemia, HSC‐derived tumors express higher levels of HSC‐associated genes including the TF Evi1 (Krivtsov *et al*, 2013). Similarly, MLL‐rearranged leukemias derived from fetal or post‐natal cells have a differential requirement for the negative regulator of E protein TFs Id1 (Man *et al*, 2016). Importantly, gene expression analysis in AML patients recently revealed that a 17‐gene “stemness” signature is highly prognostic across diverse AML subtypes and accurately predicts response to standard induction chemotherapy (Ng *et al*, 2016).

Another important factor that impacts on epigenetic heterogeneity and cell fate is the temporal sequence of mutations. Leukemogenesis occurs in a stepwise manner, with individual genetic and epigenetic perturbations accumulating over time in pre‐leukemic HSCs prior to transformation to full‐blown AML (Shlush *et al*, 2014; Morita *et al*, 2020). Historically, the phenotype of a particular cancer was considered to be the sum of the phenotypes conferred by the individual mutations within that cancer. However, as cancer genome sequencing gained sufficient depth to accurately measure variant allele frequency and infer clonal relationships, it became apparent that some mutations almost always arise in the foundation clone, whereas others typically occur later in tumor evolution (The Cancer Genome Atlas, 2013). More recently, mutation order has been shown to alter hematopoietic differentiation in humans and mice, thereby influencing not only disease progression, but also response to therapy (Ortmann *et al*, 2015; Braun *et al*, 2019; Loberg *et al*, 2019). Collectively, these studies reveal yet another pathway by which cancer evolution can lead to a bewildering number of varied outcomes. Even in two patients that carry identical genetic driver mutations, prognosis and outcomes may be different.

## Targeted manipulation of cell fate for therapeutic benefit in leukemia

Given the intimate connection between oncogenic and developmental processes, it should be possible to arrest cancer growth by permanently converting cells from a self‐renewing to a non‐self‐renewing phenotype. In essence, this is the goal of differentiation therapy. The first cancer for which differentiation therapy was demonstrated as a viable approach was acute promyelocytic leukemia (APL), a sub‐type of AML commonly driven by the t(15;17) chromosomal translocation encoding the PML‐RARα fusion protein. APL presents clinically as a massive and rapid expansion of promyelocytes in the bone marrow, with their characteristic appearance as a halfway stage between an undifferentiated “blast” and a mature granulocyte. APL cells are highly responsive to retinoic‐acid (ATRA) and arsenic trioxide (ATO), and the combination of the two compounds represents one of the true success stories of oncology, converting a once rapidly fatal disease to one that is highly curable (Lo‐Coco *et al*, 2013). The remarkable efficacy of ATRA and ATO prompted extensive efforts to understand the mechanisms that underpin their anti‐APL activity. Both compounds trigger the rapid conversion of APL cells to differentiated neutrophils *in vitro* and *in vivo*. Although the precise molecular details remain vigorously debated in the literature (Dos Santos *et al*, 2013; de Thé, 2018), at least two overlapping molecular mechanisms appear to be at play. On the one hand, ATRA has been shown to reverse the transcriptional repressor activity of PML‐RARα, enabling the reactivation of silenced target genes (Martens *et al*, 2010; Vitaliano‐Prunier *et al*, 2014). On the other hand, ATRA and ATO synergize to trigger proteolysis of PML‐RARα protein, with ATRA engaging the RARα portion and ATO engaging the PML portion (Ablain *et al*, 2013). The degradation of PML‐RARα results in the re‐establishment of PML nuclear bodies (PML‐NBs), multi‐protein structures found in many cell types that are antagonized in APL cells by the presence of the PML‐RARα fusion (Bernardi & Pandolfi, 2007). The formation of PML‐NBs, which is enhanced by the activity of ATO on the wild‐type PML protein (encoded by the remaining unaffected *PML* allele), drives senescence that is partially dependent on the TP53 axis (Ablain *et al*, 2014). Thus, while both ATRA and ATO are naturally derived compounds, the efficacy of which was discovered empirically, they are more appropriately regarded as “targeted therapies” because of their selective effects on APL.

More recently, small molecule inhibitors targeting mutant IDH proteins (IDH1 and IDH2) found in an additional 15–20% of AML patients have entered clinical use (Pollyea *et al*, 2019; Roboz *et al*, 2020). Cancer‐associated IDH mutations result in the accumulation of the oncometabolite 2‐hydroxyglutarate (2‐HG), a molecule that dysregulates many biochemical pathways including DNA, RNA and histone methylation, collagen maturation, hypoxic signaling, and DNA repair (Losman & Kaelin, 2013; Molenaar *et al*, 2018). IDH mutant proteins confer anchorage and cytokine‐independent growth and block maturation of various cell types *in vitro*, and co‐operate with additional genetic events in cancer initiation *in vivo* (Mukherjee *et al*, 2018; Zhang *et al*, 2020). Pharmacological inhibition of 2‐HG production promotes the differentiation of AML cells in model systems and patients (Losman *et al*, 2013; Kats *et al*, 2017; Shih *et al*, 2017), and though the effects on the AML differentiation hierarchy are complex and varied (Quek *et al*, 2018), these agents are able to induce complete remission (including complete molecular remission as defined by sensitive minimal residual disease assays) even in heavily pre‐treated individuals (Pollyea *et al*, 2019; Roboz *et al*, 2020).

**Figure 3:**
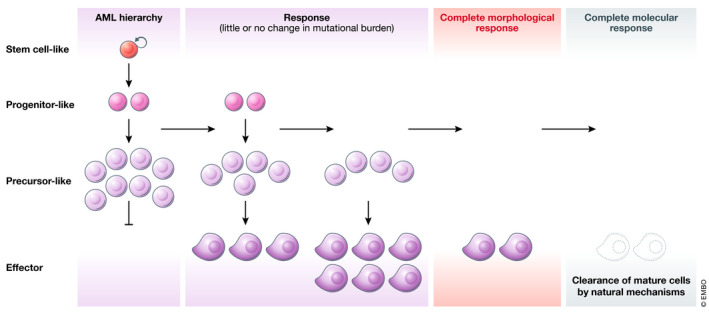
Effective differentiation therapies act at multiple points of the developmental hierarchy To achieve elimination of leukemic cells, differentiation therapies must be able to reverse the aberrant self‐renewal of leukemic stem cells (LSCs) as well as to relieve the differentiation block in leukemic blasts leading to exhaustion of the malignant hierarchy. Morphologic remission (as assessed by microscopic examination of bone marrow cells) occurs once LSCs have transited through the blast phase. Reduction and elimination of mutational burden measured by sequencing occurs later, once terminally differentiated cells have been cleared.

So, what can we learn from these two examples, and can differentiation therapy be extended to diverse AML subtypes irrespective of the underlying genetic lesions, and indeed to other cancer types? Rational engineering of novel treatment strategies requires a clear definition of the problem. Put another way, if we are seeking to reprogram cellular behavior, it is important to carefully consider the desired outcome. Differentiation therapies are classically depicted as a relief of the developmental block that prevents “blasts” from progressing to a terminally mature state. However, merely “unblocking differentiation” is insufficient unless the upstream source of the cells, the LSCs and leukemic progenitors, can also be eliminated. It is notable that in APL, morphological differentiation *per se* can be uncoupled from both self‐renewal and therapeutic efficacy, as demonstrated by treatment with low‐dose ATRA or synthetic retinoids that induce differentiation but fail to abrogate leukemogenesis (Ablain *et al*, 2013; Ablain *et al*, 2014). These studies are corroborated by clinical evidence that ATRA, a potent inducer of differentiation, is almost never curative as a single agent (Dos Santos *et al*, 2013; Vitaliano‐Prunier *et al*, 2014). Thus, for a therapy to be effective, it must be capable of normalizing the abnormal developmental trajectory of a leukemic clone and ultimately lead to its exhaustion (Fig 3).

**Figure 4:**
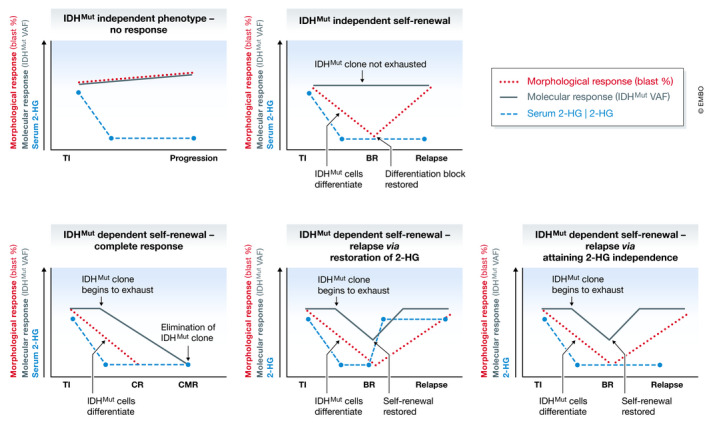
Response and resistance to inhibition of mutant IDH in AML At initiation of treatment IDH inhibitors effectively block 2‐HG production, but the effects on the phenotype of IDH mutant clones vary in different patients. Treatment efficacy is assessed by examination of the impact of the drug on hematopoiesis, with ~ 40% of patients achieving a response. In some cases, IDH inhibition induces differentiation of AML blasts, restoring normal blood development but not eliminating the mutant clone (upper right panel). In others, 2‐HG is required for self‐renewal with treatment leading to progressive depletion of mutant cells over time as LSCs differentiate and are eventually exhausted. For ~ 10% of patients, this leads to elimination of IDH mutant cells (bottom left panel). Alternatively, relapse can occur via 2‐HG dependent (bottom middle panel) and independent (bottom right panel) pathways. BR, best response (i.e., time at which the impact of the treatment on hematopoiesis has the greatest effect); CMR, complete molecular remission; CR, complete remission (normalization of hematopoiesis); TI, treatment initiation; VAF, variant allele frequency.

From a molecular perspective, gene expression programs that control self‐renewal and differentiation of AML cells are uncoupled in different cellular compartments, and consequently, the key regulators may or may not be overlapping. In some cases, an individual driver mutation may contribute to both phenotypes, as is the case in APL and a subset of IDH mutant AML. In such cases, the driver lesion is necessary to sustain the leukemic hierarchy, and directly antagonizing it using pharmacological agents can lead to complete and long‐lasting remission (Fig 4). In other cases, however, IDH mutations may be essential for the differentiation block, but dispensable for disease maintenance (Fig 4). In these patients, IDH mutant clones undergo terminal differentiation in response to IDH inhibition, and though the patients may enter a morphological complete remission, the clones are not extinguished and lead to relapse if the differentiation block is restored by subsequent genetic events (Quek *et al*, 2018). The reasons for these divergent outcomes remain the subject of intense study. In a proportion of relapsing patients, gatekeeper mutations or isoform switching (i.e., the acquisition of an IDH1 mutation in an IDH2 mutant clone or vice versa) results in re‐establishment of 2‐HG production, the essential downstream mediator of mutant IDH proteins (Harding *et al*, 2018; Intlekofer *et al*, 2018). In most cases however, relapse from an IDH mutant clone occurs in spite of continued suppression of 2‐HG, indicating that the clone has become mutant IDH‐independent (Fig 4) (Amatangelo *et al*, 2017; Quek *et al*, 2018). Although some clinical observations have linked the acquisition of certain mutations (e.g., NRAS) with resistance to mutant IDH inhibitors, genetic changes cannot easily account for the escape from 2‐HG addiction in most patients, suggesting epigenetic mechanisms (Quek *et al*, 2018). Going forward, functional validation in physiologically relevant model systems that recapitulate the developmental complexity of AML is needed.

Beyond targeting essential oncogenes, different AML mutations likely converge on a limited number of molecular nodes that govern cell fate transitions. Targeting such nodes represents an attractive alternative to targeting the driver mutations themselves and in principle would yield therapies that could be broadly deployed against AML. Critical pathways that sustain LSCs have been reviewed extensively elsewhere (Pollyea & Jordan, 2017) and include metabolic enzymes and epigenetic regulators that are amenable to small molecule inhibition. One prominent example is the MLL1–Menin complex that has emerged as a targetable entity in MLL‐rearranged and NPM1‐mutant AML. Although initiated by genetic perturbations that are seemingly unrelated, both subtypes are characterized by self‐renewal within committed myeloid progenitors that is dependent on high expression of *HOXA* genes and can be abrogated by interruption of the MLL1–Menin interaction (Klossowski *et al*, 2020; Uckelmann *et al*, 2020). Furthermore, the application of genetic and compound screens that use differentiation as a readout (as opposed to viability or proliferation) has recently yielded a number of other promising and unexpected targets including GSK‐3α, DHODH, and LSD1 (Banerji *et al*, 2012; Sykes *et al*, 2016; Bell *et al*, 2019). A number of inhibitors of these pathways are now in clinical development and are likely to increase therapeutic options in AML in the short‐to‐medium term.

## The implications of plasticity for drug resistance and anti‐cancer immunity

Although the existence of a hierarchy in AML and most other malignant blood disorders during disease development is well accepted, significant evidence has emerged that cell fate in leukemia is highly malleable in response to extrinsic pressure. Pre‐existing non‐genetic heterogeneity and the capacity of cancer cells to reprogram their developmental trajectories are fertile grounds for the emergence of drug resistance (Bell & Gilan, 2020). A variety of mechanisms have been described that allow malignant cells to maintain both proliferation and self‐renewal, enabling the disease to overcome various insults imposed by therapies.

Acute lymphoid leukemia can undergo lineage switching and adopt a myeloid phenotype in response to immune agents such as antibodies and CAR‐T cells targeted against the lymphoid marker CD19 (Gardner *et al*, 2016; Jacoby *et al*, 2016). Mechanistically, this is mediated by increases in chromatin accessibility of myeloid TF motifs at the expense of lymphoid TF motifs (Jacoby *et al*, 2016). Interestingly, the transient priming of lymphoid TF motifs has been reported within committed myeloid progenitors during normal blood formation (Buenrostro *et al*, 2018). It is therefore tempting to speculate that LSCs committed to lymphoid differentiation may exhibit transient expression of a myeloid program in an analogous manner, thereby facilitating immune escape.

The traditional CSC model posits that conversion between self‐renewing tumorigenic cancer cells and differentiated cancer cells with limited proliferative potential is unidirectional. Recent studies have disputed that notion however, demonstrating that even mature granulocytes are not beyond being able to dedifferentiate and reinitiate disease in certain contexts (McKenzie *et al*, 2019). Transition of leukemic cells to a state capable of regeneration has been implicated in driving resistance to various agents including BRD4 inhibitors and cytotoxic chemotherapy (Fong *et al*, 2015; Farge *et al*, 2017; Boyd *et al*, 2018; Bell *et al*, 2019). These studies suggest that rather than selecting for cells with pre‐existing LSC function, surviving cells adopt that capability and emerge following drug pressure. Notably, understanding these adopted states, which are often transient, is critical to the design of “developmental traps” that akin to “evolutionary traps” seek to exploit targetable vulnerabilities that emerge in a predictable manner.

The multitude of phenotypes expressed by an individual cancer genetic clone affects its interaction with the microenvironment and the immune system. A recent study by Paczulla *et al* found that functionally defined LSCs, but not differentiated leukemic cells, lack expression of NKG2D ligands that are recognized by natural killer (NK) cells. Lack of NKG2D expression protects LSCs from NK‐mediated killing and contributes to their ability to maintain disease in an immune‐competent host (Paczulla *et al*, 2019). Conversely, leukemic hierarchies in some patients comprise large populations of mature AML cells that resemble myeloid‐derived suppressor cells (van Galen *et al*, 2019). These cells can inhibit T‐cell proliferation *in vitro*, and their presence is correlated with reduced T‐cell infiltration *in vivo* and likely contributes to poor immune clearance of malignant cells. The observations that AML developmental hierarchies impact on anti‐cancer immune responses have significant therapeutic implications, particularly in light of the growing availability and deployment of immuno‐oncology agents. There are reasons to speculate that T‐cell activity is crucial in clearing minimal residual disease in AML and preventing disease relapse (Teague & Kline, 2013). However, accumulation of differentiated myeloid cells as a consequence of differentiation therapies may enhance the T‐cell suppressive microenvironment and promote leukemic persistence. It may therefore be necessary to combine such agents with immune‐modulatory modalities that are capable of boosting T‐cell responses.

The differentiation state of a leukemic clone, both during leukemogenesis and in response to targeted therapies, may also influence the phenotype of genetically distinct clones in the same niche. A notable and not uncommon observation is that AML patients with a sub‐clonal IDH mutant disease can nonetheless enter complete remission in response to IDH inhibitor therapy (Quek *et al*, 2018). Although not formally tested, the response of IDH wild‐type cells is likely to be the result of paracrine effects induced by IDH mutant cells with communication between different genetic clones occurring through secreted factors or direct cell‐to‐cell contact.

## Evidence and application in solid malignancies

In the past two decades, knowledge of adult stem cells in various non‐hematopoietic tissues has grown considerably. Novel conditional mouse models that enabled definitive lineage tracing and/or selective depletion of defined cell types, as well as advancements in organoid cultures, have revolutionized our understanding of how different organs develop, are sustained, and regenerate following injury. An emerging theme is that classical features of blood development, in particular the preeminent and unique role of HSCs in life‐long maintenance of hematopoiesis, are not generalizable to all solid organs (Post & Clevers, 2019). Although stem cell function is bestowed on a small number of “professional” cells in some contexts such as skeletal muscle, in other tissues many cells can assume stemness, especially in response to injury. In the intestinal crypt, for example, Lgr5^+^ stem cells can be replaced by lineage‐committed progenitors through plasticity and interaction with the stem cell niche (Tetteh *et al*, 2016). In the liver, the situation appears to be even more extreme, with some studies suggesting that organ regeneration is driven by the proliferation and trans‐differentiation of differentiated cells rather than “pre‐defined” stem cells (Raven *et al*, 2017). These findings suggest that mammalian evolution has developed different strategies for replacing lost cells that likely reflect the distinctive needs of specific tissues.

The inherent plasticity of certain lineages presents challenges, but also opportunities, for the development of differentiation therapies, and indeed therapies more broadly. Even an individual cancer genetic clone is a complex ecosystem comprised of diverse cell types, each with unique dependencies and sensitivities. Recent single‐cell analyses have laid bare the epigenetic complexity of solid tumors, enabling “unbiased” classification of individual cells based on a large number of features (as opposed to a few pre‐defined cell surface markers) (Tirosh *et al*, 2016; Praktiknjo *et al*, 2020). Advanced computational methods such as pseudo‐temporal ordering have allowed the inference of relationships between different cellular states (Trapnell *et al*, 2014). Encouragingly, it may be possible to collapse the malignant hierarchy in some tumors as exemplified by targeting R‐spondins in colon tumors driven by the PTPRK‐RSPO3 fusion protein (Storm *et al*, 2016). Likewise, our capacity to identify rare drug‐persistent cells that survive treatment and are the substrate for genetic evolution that eventually leads to resistance has led to elegant strategies to circumvent relapse before it can occur (Liau *et al*, 2017; Sharma *et al*, 2018; Rambow *et al*, 2018). Collectively, these and other studies offer hope that increased knowledge of cellular behavior can improve patient outcomes.

## Future directions and conclusions

Non‐genetic heterogeneity and plasticity stand alongside genetic variation as major factors that affect the outcome of anti‐cancer therapy. Genetic cancer clones express variable phenotypes, and in many contexts are endowed with distinct capability to maintain tumor growth. Many therapies are able to target some but not all aspects of this complex ecosystem and may promote cells to enter novel developmental states. If the organization of the malignant clone is hierarchical, therapies that promote the exhaustion of the clone by converting self‐renewing cancer cells to a non‐proliferative state can be extremely effective, as exemplified by ATRA/ATO in APL. Importantly though, even if cell fate is plastic, as it is in many solid cancers, the potential paths that non‐self‐renewing cells can take to acquire regenerative potential in response to selective pressure may be limited and predictable. Understanding these processes will aid in the design of novel strategies that target transient vulnerabilities when the cancer is at its weakest point and prior to the inevitable tsunami of genetic changes that may render it uncontrollable.

**Pending issues**

- How particular molecular features (e.g., gene or protein expression, chromatin state, genotype) influence cellular behavior in specific contexts requires further investigation. Until recently, analyses have been limited by the number of features that could be simultaneously measured at single‐cell level. Advances in single‐cell omics have greatly increased our ability to differentiate between relative changes in cell frequency within a population and changes within specific cell types that could not be achieved by measurements of population averages. Moreover, our capacity to multiplex different types of measurements is providing powerful insights into the interplay between genetic and epigenetic heterogeneity.
- A greater understanding of spatial heterogeneity is required. Emergence of spatial transcriptomics will greatly advance our knowledge of the interaction between different malignant and non‐malignant cells.
- As promising non‐cytotoxic therapies that modulate differentiation and self‐renewal (e.g., inhibitors of IDH1/2, LSD1, DHODH) and LSC‐directed strategies specifically targeting the subset of cells required to maintain AML (e.g., Venetoclax/Azacitidine) enter clinical use, we must continue to learn from both successes and failures. Correlative observations in patients can be used to generate hypotheses that can be tested in model systems, with the ultimate goal of expanding the success of differentiation therapy beyond APL to other cancer contexts.

## Conflict of interest

LMK has received research funding and consultancy payments from Agios Pharmaceuticals and Celgene Corporation.
